# Metabolic reprogramming in the tumor microenvironment of liver cancer

**DOI:** 10.1186/s13045-024-01527-8

**Published:** 2024-01-31

**Authors:** Jian Lin, Dongning Rao, Mao Zhang, Qiang Gao

**Affiliations:** 1 Center for Tumor Diagnosis and Therapy, Jinshan Hospital, Fudan University, Shanghai, China; 2 Department of Liver Surgery and Transplantation, Liver Cancer Institute, Zhongshan Hospital, Key Laboratory of Carcinogenesis and Cancer Invasion of Ministry of Education, Fudan University, Shanghai, 200032 China; 3 Key Laboratory of Medical Epigenetics and Metabolism, Institutes of Biomedical Sciences, Fudan University, Shanghai, 200032 China; 4 Zhejiang Provincial Key Laboratory of Pancreatic Disease, The First Affiliated Hospital, Zhejiang University School of Medicine, Hangzhou, China

**Keywords:** Liver cancer, Metabolic reprogramming, Immune microenvironment, Gut–liver axis

## Abstract

The liver is essential for metabolic homeostasis. The onset of liver cancer is often accompanied by dysregulated liver function, leading to metabolic rearrangements. Overwhelming evidence has illustrated that dysregulated cellular metabolism can, in turn, promote anabolic growth and tumor propagation in a hostile microenvironment. In addition to supporting continuous tumor growth and survival, disrupted metabolic process also creates obstacles for the anticancer immune response and restrains durable clinical remission following immunotherapy. In this review, we elucidate the metabolic communication between liver cancer cells and their surrounding immune cells and discuss how metabolic reprogramming of liver cancer impacts the immune microenvironment and the efficacy of anticancer immunotherapy. We also describe the crucial role of the gut–liver axis in remodeling the metabolic crosstalk of immune surveillance and escape, highlighting novel therapeutic opportunities.

## Background

Primary liver cancer (PLC) is one of the most common malignancies worldwide, with hepatocellular carcinoma (HCC) accounts for 75–85% and intrahepatic cholangiocarcinoma (iCCA) for 10–15% (Other rare PLCs are not discussed in this Review) [1]. The heterogeneous nature of PLC challenges the development of new treatment strategies, especially for patients at advanced stage. Systemic agents, such as multi-kinase inhibitors, have achieved great progress since 2007, and combined anti-vascular endothelial growth factor (VEGF) or tyrosine kinase inhibitors (TKIs) with immune checkpoint blockades (ICBs) has been endorsed as the new standard of care in first-line treatment for advanced HCC [2]. The ICB-based regimen has also obtained impressive survival benefits in two recent phase 3 trials for the treatment of advanced biliary tract cancer, including iCCA [3, 4]. However, most of these treatment options provide limited extensions of overall survival, often with miserable life qualities. A better understanding of the complex microenvironment within PLC may help in developing novel therapeutic interventions to augment immune-based therapies.

While the global incidence of hepatic virus B or C (HBV or HCV)-related PLCs is decreasing due to successful interventions of vaccinations or anti-viral treatments, obesity-related disorders and associated morbidity/mortality are continuously rising, especially in Western countries [2]. The liver is not only an immune-privileged organ with a high tolerance for gastrointestinal tract-derived antigens, but also the central hub for various metabolic processes and the maintenance of homeostasis. This underscores the importance of metabolic rearrangements during hepatic carcinogenesis [1]. Increasing evidence reveals that the liver receives gut bacterial metabolites through the blood supply from the intestine. Changes in the gut microbiome disturb immune cell infiltration and function in the tumor microenvironment (TME) of PLC, potentially affecting the efficacy of current immunotherapies [5].

Metabolism, a fundamental biological process of a living cell, converts nutrients to generate extensive energy (i.e., ATP), redox equivalents (i.e., NADPH), and macromolecules (i.e., lipids, proteins, and nucleic acids). Tumor cells often exhibit a high dependence on a reprogrammed metabolic state for stress adaptation and infinite proliferation, which can be leveraged for non-invasive cancer diagnosis [6–8]. Unlike normal cells, which usually acquire energy through oxidative phosphorylation (OXPHOS), tumor cells tend to choose glycolysis even in the presence of oxygen, also known as “Warburg effect” [9]. Although aerobic glycolysis is way more inefficient than OXPHOS (2 ATP vs. 36 ATP per glucose), the ATP production rate of aerobic glycolysis is much higher, which better satisfies the greedy reproduction of neoplastic cells [10]. On the other hand, excessive lactate generated by aerobic glycolysis also fuels neighboring oxygenated cells, leading to a metabolic symbiosis between glycolytic and oxidative metabolism [11–13]. Due to significant glucose uptake in PLC bulk tissues, ^18^F-2-deoxyglucose (^18^FDG) positron emission tomography (PET) imaging is widely used for PLC diagnosis and progression monitoring [14]. In addition to glucose metabolism, other central metabolic pathways are often reprogrammed, leading to dysregulated nutrient depletion, oncometabolite accumulation, and signaling pathway perturbations in the TME [15].

The metabolic changes during PLC progression contribute to identifying pathogenic mechanisms and therapeutic targets and developing novel prognostic and diagnostic biomarkers. Just like the TME, liver cancer metabolism is heterogeneous, encompassing metabolic signatures from tumor, stromal, and immune cells. Emerging evidence indicates that metabolic alterations in tumor cells affect the composition and function of surrounding cells [16, 17]. With the advent of immunometabolism in cancer treatment [18–22], greater attention should be given to the interplay of metabolism-related immune signaling. In this review, we focus on how metabolically rewired liver tumor cells cultivate an immunosuppressive microenvironment through tumor immune metabolic interactions. We also discuss the impact of the gut–liver axis on the liver microenvironment and ICB-based immunotherapies. The purpose of this review is to link recent findings on the crosstalk between liver cancer metabolism and immunometabolism, potentially revealing novel therapeutic opportunities.

## General characteristics of liver cancer metabolism

**Fig. 1:**
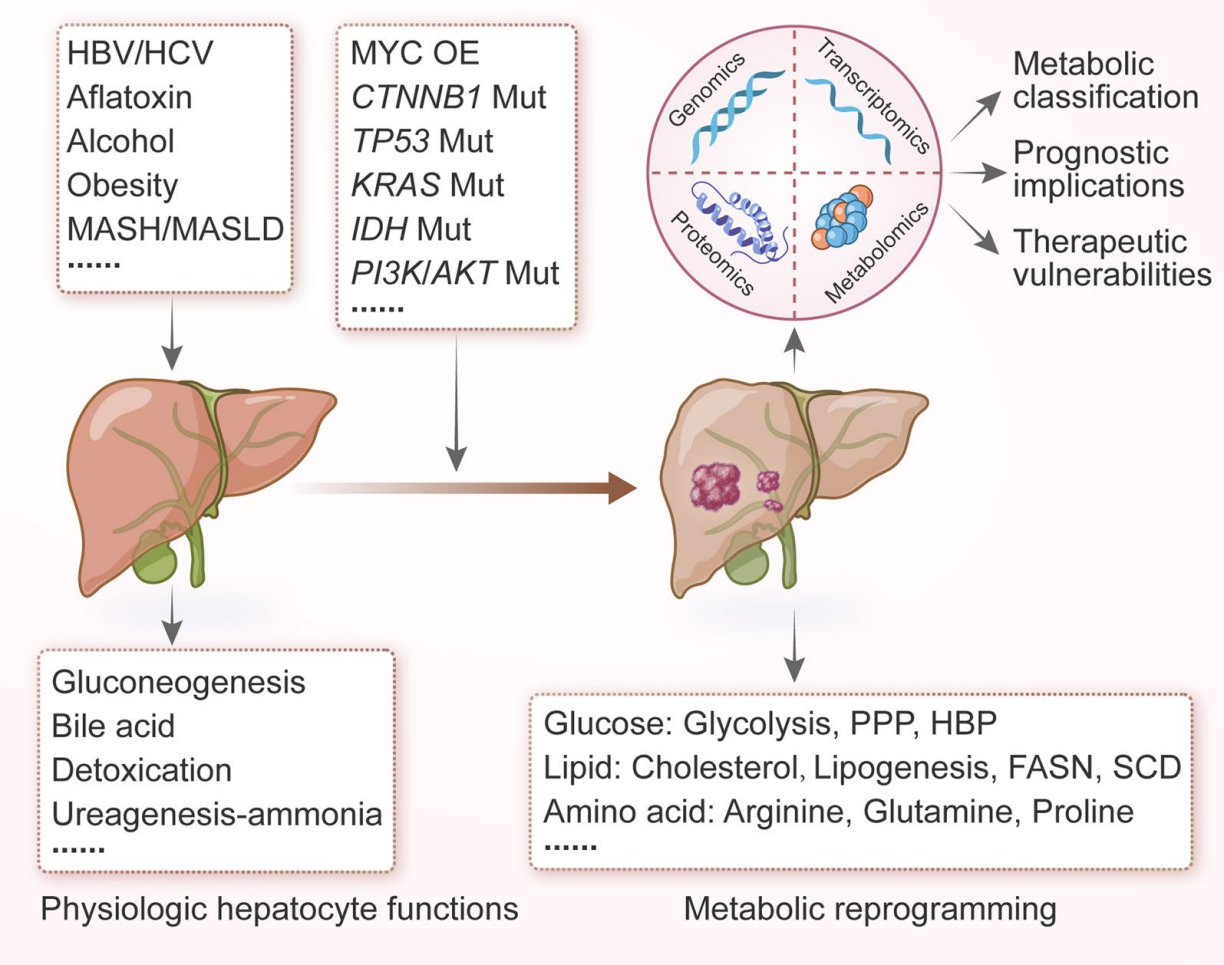
Metabolic alterations in PLC. HBV: hepatitis B virus; HCV: hepatitis C virus; MASH: metabolic dysfunction-associated steatohepatitis; MASLD: metabolic dysfunction-associated steatotic liver disease; OE: overexpression; Mut: mutation; PPP: pentose phosphate pathway; HBP: hexosamine biosynthesis pathway; FASN: fatty acid synthase; and SCD: stearoyl-CoA desaturase

The liver is the largest internal organ for controlling metabolism, and metabolic disruption is closely associated with hepatocarcinogenesis. Common risk factors for PLC include HBV/HCV infection, alcohol abuse, obesity, metabolic dysfunction-associated steatohepatitis (MASH), and metabolic dysfunction-associated steatotic liver disease (MASLD). In addition, exposure to aflatoxin promotes the development of HCC, and liver fluke infection, biliary duct cysts, and primary sclerosing cholangitis (PSC) are established risk factors for iCCA [1]. These risk factors may promote the initiation and progression of PLC through metabolic reprogramming [1, 23]. Therefore, understanding metabolic alterations in liver cancer is vital for identifying pathogenic mechanisms and exploring therapeutic targets (Fig. 1).

Metabolic classification aids in understanding the heterogeneous metabolic microenvironment and developing personalized interventions. Systemic analysis using multi-omics has been crucial in outlining the varied metabolic landscape of HCC. By integrating genomics, transcriptomics, and proteomics data retrieved from several public datasets, genome-wide metabolic models (GEMs) stratified HCC patients into three prognostic subgroups with significant differences in dysregulated kynurenine metabolism (iHCC1), WNT/β-catenin-related lipid metabolism (iHCC2), and PI3K/AKT/mTOR signaling (iHCC3) [24]. Consistent with the histological features [25], both iHCC1 and iHCC2 exhibit hepatocyte differentiation and maturation, whereas iHCC3 is associated with proliferation and immune activation [24]. Multi-omics studies have nominated metabolic pathways as the most dramatic alterations in HCC and iCCA, compared with normal adjacent tissues [26–29]. During liver cancer progression, typical hepatocyte metabolic functions, such as gluconeogenesis, bile acid (BA) metabolism, detoxication, and ureagenesis-ammonia, are diminished. This decline is accompanied by an increase in tumor malignancy [30, 31], most likely due to the de-differentiation from functional hepatocytes to HCC cells. Recently, we identified two metabolic subtypes in 65 human liver cancer organoids through multi-omics profiling, which complements our understanding of HCC tissue metabolism. Glucose-6-phosphate dehydrogenase (G6PD) was identified as a potential target in the subtype with an enriched drug metabolism pathway, consistent with the previous results [32–35]. Meanwhile, classifying patients based on a single metabolic pathway can guide personalized therapy. For instance, HCC patients were categorized into three metabolic subtypes (F1, F2, and F3) based on the expression pattern of 42 fatty acid degradation (FAD) genes, revealing distinct clinical/molecular characteristics and treatment vulnerabilities. Interestingly, the F1 subtype with the lowest expression levels of FAD genes shows a high degree of immune infiltration, designated “hot” tumor. Thus, HCC mouse models derived from the F1 subtype are responsive to anti-PD-1 (programmed cell death protein 1) therapy, in contrast with mouse models derived from the F2 and F3 subtypes [36]. Taken together, systems biological approaches in metabolic signature deconvolution can illuminate metabolic heterogeneity and identify potential metabolic targets for PLC (Fig. 1).

Systemic analysis focusing on metabolic gene expressions and non-targeted metabolic profiling has shown that aerobic glycolysis, lipid metabolism, and amino acid metabolism are the main metabolic alterations in HCC tissues [31, 37]. Cancer cells often face hypoxic and hypo-nutrient environments, necessitating metabolic rearrangement to satisfy energy demands and biomass synthesis. It has been demonstrated that liver cancer cells typically utilize glycolysis under hypoxic conditions to produce lactate via lactic dehydrogenase (LDH) [38]. In addition to creating an acidic microenvironment that promotes tumor progression, lactate accumulation also causes lactylation of adenylate kinase 2 (AK2) at K28, compromising its kinase activity and disrupting energy homeostasis in HCC cells, thereby facilitating tumor proliferation, invasion, and metastasis as shown in several xenograft mouse models [39]. Based on several retrospective analyses [40–42], high serum LDH levels are associated with poor prognosis after curative resection or standard therapies in both HCC and iCCA. Hypoxia also induces micropinocytosis for nutrient scavenging via the hypoxia-inducible factor (HIF)/EH domain-containing protein 2 (EHD2) pathways in several HCC cell lines and mouse models [43]. Other glucose metabolic pathways, such as the pentose phosphate pathway (PPP) and the hexosamine biosynthetic pathway (HBP), are also more active in HCC tissue compared to normal adjacent tissues [44–47] (Fig. 1).

In high-fat diet (HFD)-induced HCC, or steatohepatitic HCC, the fatty acid oxidation (FAO) pathway tends to be downregulated to protect HCC cells from lipotoxicity [48]. Concordantly, de novo lipogenesis gradually increases from normal liver tissue to liver tumors and is generally associated with advanced HCC and worse patient prognoses [49–52]. However, an independent study in a diethylnitrosamine (DEN)-induced mouse model showed that liver-specific knockout of acetyl-CoA carboxylase (ACC) inhibiting de novo lipogenesis accelerates HCC progression by activating antioxidant defense. This discrepancy may be attributed to differences between clinical samples and preclinical models of HCC, highlighting the need for a thorough exploration of tumor-driving events and metabolic plasticity [53]. Lipid metabolic pathways, including fatty acid synthase (FASN) and stearoyl-COA desaturase (SCD) signaling, also sustain cancer stem cells in HCC, contributing to metastasis and drug resistance [54]. High-cholesterol diets induce HCC in mice, partly through dysregulation of metabolism and calcium signaling [55–57]. Integrated proteomics and phosphoproteomics have revealed that targeting sterol O-acyltransferase 1 (SOAT1) to reduce cholesterol content in plasma membranes presents effective treatment options for early-stage HCC patients, which was further verified in a patient-derived tumor xenograft mouse model [58]. Conversely, high serum cholesterol levels are linked with better patient outcomes by inhibiting tumor metastasis [59], implying that cholesterol distribution and homeostasis significantly influence HCC tumorigenesis (Fig. 1).

Moreover, numerous studies indicate enhanced amino acid metabolism in liver tumors compared to non-tumor tissues [1, 31, 37, 60, 61]. Sustained urea cycle repression in liver cancer shifts metabolism from arginine production to pyrimidine biosynthesis. HCC cells depend on external arginine sources, with arginine restriction inducing a general control nonderepessible 2 (GCN2) kinase-related stress response. GCN2 suppression leads to cell senescence and increases sensitivity to senolytic treatment both in vitro and in vivo. Thus, combining GCN2 inhibition with senolytic agents could be an effective treatment strategy in arginine-deprived HCC cells [62]. In an mTOR-driven HCC mouse model, tumor cells also increased arginine import and reduced its conversion to polyamines, driving oncogenic metabolism via the arginine-binding factor RNA-binding motif protein 39 (RBM39). Targeting RBM39 instead of circulating arginine may offer a way to reverse the oncogenic pathway triggered by high arginine pools in HCC cells, thereby avoiding the adverse side effects of circulating arginine-depleting therapy [63]. Glutamine, the most abundant amino acid in human blood, is a key carbon source for de novo lipogenesis in mitochondrial dysfunctional HCC [64]. Glutamine addiction in glutamine synthetase (GS)-overexpressing HCC supports mTOR-dependent cell proliferation and survival in clinically relevant HCC models [65]. Additionally, the glutamate-to-proline biosynthetic flux is elevated in tumor tissues, promoting HCC cell proliferation and tumor growth in both tumor models and regenerating tissues [66]. Folate-mediated one-carbon (1C) metabolism contributes to the availability of various building blocks for tumor cell proliferation [67–70], and the expression of central enzymes involved in 1C metabolism is largely dysregulated in PLC [71, 72]. Serine, glycine, and methionine metabolism is tightly linked to the generation of 1C units. In HCC cells, glycine-derived 1C units support purine and pyrimidine biosynthesis and tumor progression through glycine cleavage system (GCS) flux [73]. Recent findings have also highlighted the promotion of tumor development by dietary folate supplementation through the integration of methionine and 1C metabolism in the HCC mouse model induced by DEN/HFD [74].

We and others have identified numerous putative driver genes that reshape PLC metabolism [29, 30, 75–79] (Table 1). The proto-oncogene *Myc* is overexpressed in nearly 70% of viral and alcohol-related HCC. Studies in HCC cell lines show that MYC overexpression upregulates glucose transporters GLUT1 and GLUT2, hexokinase HK2, and pyruvate kinase isoforms PKL/PKM, thereby enhancing tumor glycolysis. High levels of GLUT1 expression are also associated with poor prognosis in both HCC and iCCA [80–84]. Under glucose/glutamine-deprived conditions, overexpressed cMYC also activates the serine biosynthesis pathway to adopt metabolic switch through transcriptionally upregulated the final rate-limiting enzyme phosphoserine phosphatase in both HCC cell lines and xenograft mouse models [85]. Furthermore, the “Warburg effect” is promoted by *TP53* mutations and PI3K/AKT/mTOR pathway activation in PLCs through upregulating related glycolytic enzymes [86, 87]. Wnt-β-catenin signaling is often hyperactivated, promoting PLC growth and dissemination [88]. β-catenin (encoded by *CTNNB1*) oncogenic activation in HCC cells induces FAO through the transcription factor peroxisome proliferator-activated receptor α (PPARα) [89]. Additionally, *CTNNB1*-mutated HCC cells rely on glutamine synthetase-dominated mTORC1 signaling for metabolism [65]. In an iCCA patient cohort, KRAS alterations lead to GLUT1-mediated glycolysis and poor patient outcomes [90]. Significant alterations in metabolic genes, including *ALB*, *APOB*, and *IDH1/2*, also induce metabolic changes in PLC [77, 91]. Leveraging genetically engineered mouse models of iCCA, *IDH* mutations were shown to increase the production of D-2-hydroxyglutarate (D-2-HG), affecting α-ketoglutarate (αKG)-dependent dioxygenases involved in DNA repair and epigenetic remodeling [92]. Thus, oncogenic alterations can also drive metabolic rearrangements in PLC (Fig. 1).

**Table 1:** Main oncogenic drivers and associated metabolic alterations in PLC

Oncogenic drivers	Target molecules	Dysregulated metabolic pathways	PLC types (proportions)	References
MYC OE	GLUT1/2	Glycolysis	HCC (15%)	[82, 83, 85]
	HK2	Serine biosynthesis	iCCA (6%)	
	PKL/PKM			
	PSPH			
*TP53* mut	GLUT1/4	Oxidative glycolysis	HCC (58%)	[86, 90]
			iCCA (21%)	
*CTNNB1* mut	PPARα	FAO	HCC (19%)	[65]
	GS	mTORC1		
*APOB* mut		VLDL secretion	HCC (10%)	[77]
*ALB* mut		Albumin production	HCC (9%)	[78]
*KRAS* mut	GLUT1	Glycolysis	iCCA (17%)	[90]
*IDH1/2* mut	αKG-dependent dioxygenases	TCA	iCCA (17%)	[92]
*BAP1* mut	Histone H2A and mitochondrial ubiquitination	Gluconeogenesis and lipid homeostasis	iCCA (12%)	[79]

*OE* overexpression; *Mut* mutation; *GLUT* glucose transporter; *HK2* hexokinase 2; *PKL/PKM* pyruvate kinase isoforms L/M; *PSPH* phosphoserine phosphatase; *PPARα* peroxisome proliferator-activated receptor-α; *GS* glutamine synthetase; *αKG* α-ketoglutarate; *FAO* fatty acid oxidation; *VLDL* very low-density lipoprotein; and *TCA* tricarboxylic acid

## Metabolic interactions between tumor cells and the TME

The cancer-immunity cycle (CIC) describes the consecutive anti-tumor responses of the immune system, including the release of cancer cell antigens, the presentation of cancer-associated antigens, the priming and activation of T-cell, and their trafficking to the tumor site, followed by infiltration into the tumor and stroma, recognition of the tumor cells, and, ultimately, killing of the tumor cells [93]. High metabolic turnover is the typical feature of rapidly proliferating and differentiating cells. Metabolic derangements within the TME are increasingly recognized as one of the most important factors halting the CIC [94]. Tumor cells orchestrate metabolism to meet their prodigious anabolic demands, creating a microenvironment featured by hypoxia, acidification, and essential nutrient depletion for adjacent immune cells. Generally, intense nutrient competition occurs between tumor cells and anti-tumor immune cells [18]. Glucose, amino acids, and fatty acids are critical energy sources not only for tumors, but also for proliferative cells, particularly anti-tumor immune cells [95]. Tumor cells often extract these nutrients from the tumor interstitial fluid (TIF) to hinder tumorolytic activities [10, 16, 96–101]. Meanwhile, the aberrant consumption of macromolecules and metabolic substances leads to the production of numerous byproducts, and some of them could be harmful to immunosurveillance [10]. Oncometabolites, such as lactic acid [102–114], kynurenine [115–117], adenosine [117], 2-HG [118, 119], and prostaglandin E2 (PGE2) [120], typically antagonize the anti-tumor immune response and/or promote the immunosuppressive activities of TME components, ultimately leading to immune evasion. Currently, the monocarboxylate transporter MCT1 inhibitor AZD3965 (for lactate symporter) [121], the indoleamine 2,3-dioxygenase IDO1 inhibitor Indoximod/Epacadostat (for kynurenine synthesis) [122], the CD73 inhibitor Oleclumab (for adenosine conversion) [123], the isocitrate dehydrogenase IDH1/2 inhibitor Enasidenib/Ivosidenib (for 2-HG production) [124], and the cyclooxygenase COX-2 inhibitor Celecoxib (for PGE2 accumulation) [125, 126] are being evaluated or approved to target cancer metabolism, aiming to enhance the efficacies of current therapies [127].

Metabolically reprogrammed tumors can foster an immune-suppressed TME by modulating the expression of signaling molecules such as immune checkpoints, chemokines, cytokines, etc. On the other hand, the pro-tumor attributes trigger dysregulated signaling or metabolic pathways, leading to the metabolic reprogramming of immune cells. Notably, anti-tumor immune cells often exhibit metabolic profiles complementary to their pro-tumor counterparts. For instance, immune-activated cells, including effector T (Teff) cells, nature killer (NK) cells, dendritic cells (DCs), and inflammatory tumor-associated macrophages (TAMs), primarily exhibit high glycolysis activity. In contrast, immunosuppressive cells such as regulatory T (Treg) cells, TAMs, and myeloid-derived suppressor cells (MDSCs) typically rely on OXPHO or FAO to sustain their function [18] (Table 2). This metabolic heterogeneity underpins the immunosuppressive TME and supports the unrestrained growth of tumor cells.

**Table 2:** Metabolic features of immune cells in the TME

Cell types	Metabolic characteristics					
	Glycolysis	OXPHOS	FAO	AA	PPP	HBP
*Immune activation*						
Teff	+ +	+		+	+	+
Tem	+ +	+				
NK	+	+				
M1φ	+ +				+	
DC	+ +					
*Immunosuppression*						
Treg		+ +	+	+		
MDSC	+		+ +	+		
M2φ		+	+ +	+		

+  + Significantly upregulated; + Upregulated; *OXPHOS* oxidative phosphorylation; *FAO* fatty acid oxidation; *AA* amino acid; *PPP* pentose phosphate pathway; and *HBP* hexosamine biosynthetic pathway

### Nutrient competition in liver cancer

Glutamine metabolism is essential for both proliferative cancer cells and activated CD8 + T cells. In the TCGA HCC cohort, glutamine metabolism-related gene expression scores inversely correlate with patient prognoses. In the glutamine-dominant HCC subgroup, CD8 + Teff cells shift to metabolizing exogenous lipids due to limited access of glutamine, reducing their quantity and cytolytic function [128]. In vitro co-culture assays have indicated that glutamine deprivation in the TME induces mitochondrial damage and CD8 T-cell apoptosis, impairing their tumorolytic function [129]. Apart from glutamine, glucose is critical for the metabolic fitness of tumor-infiltrating cytotoxic CD8 + T cells [18]. The results of a recent clinical trial demonstrated the promising efficacy of combined IFNα and ICB therapy in patients with advanced melanoma [130]. Our group further demonstrated that IFNα therapy could strongly enhance the efficacy of ICB in both patients with HCC and preclinical HCC mouse models. Mechanistically, IFNα therapy inhibits HIF1α signaling to reduce glucose consumption in tumor cells. The consequent accumulation of glucose in the TME stimulates the expression of the costimulatory molecule CD27 via mTOR–FOXM1 signaling in CD8 + T cells, thereby reinforcing the functions of cytotoxic T cells in both immunocompetent orthotopic and spontaneous HCC models [131].

**Fig. 2:**
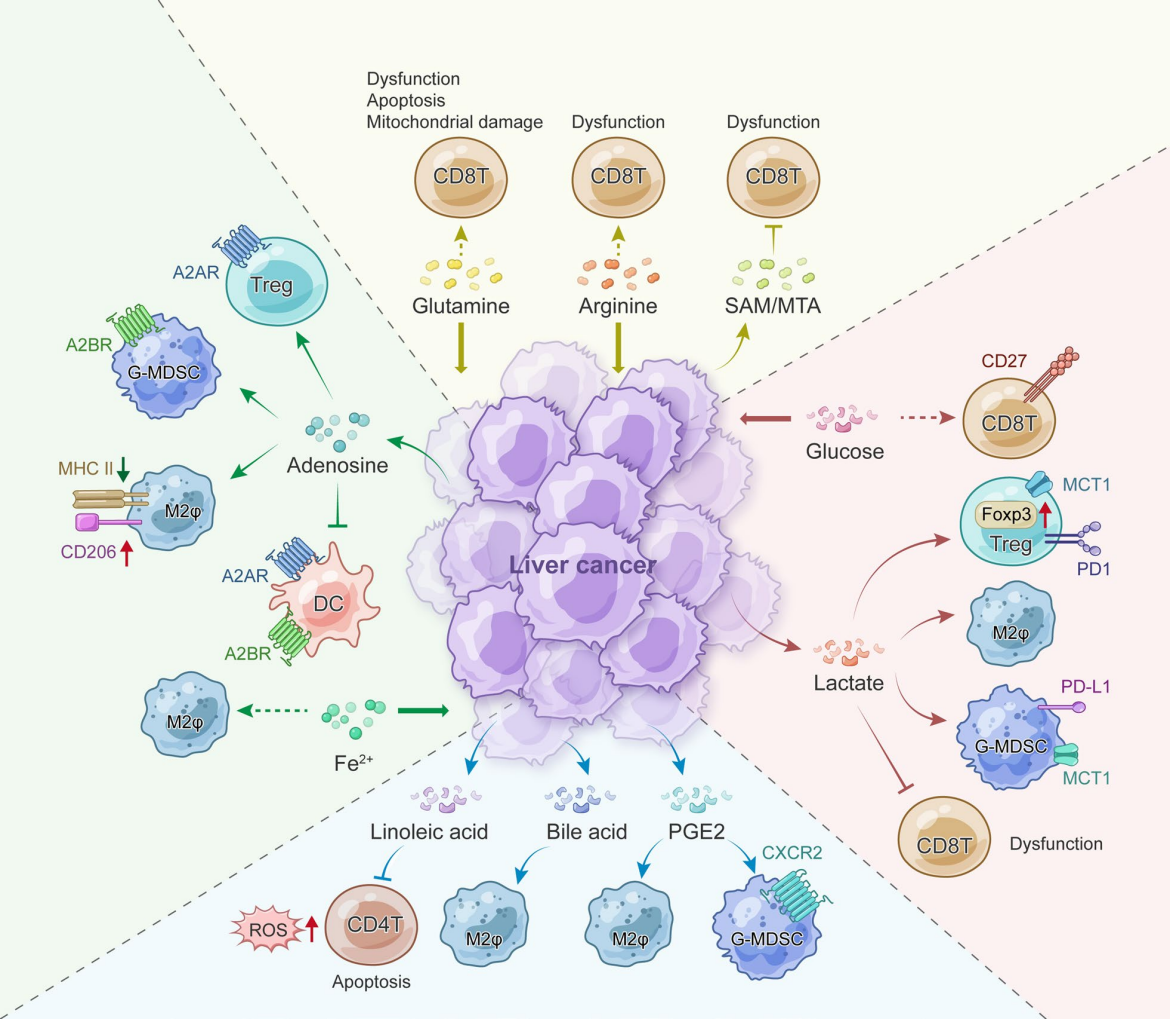
Immune regulation by nutrient competition and oncometabolite production. SAM: S-adenosylmethionine and MTA: 5-methylthioadenosine

Nutrient depletion in liver cancer also influences the shift from anti-tumor M1-like macrophages (M1φ) to pro-tumor M2-like macrophages (M2φ). Compared to M1φ, M2φ tends to polarize under low ferrous iron levels [132]. The hypoxic HCC microenvironment prompts tumor cells to compete with macrophages for iron through increased transferrin receptor (TFRC) expression, the primary receptor for transferrin‑mediated iron uptake. This iron competition culminates in an M2-like TAM polarization *in vitro* [133]. Taken together, nutrient competition between liver cancer cells and immune cells can either weaken anti-tumor immunity or enhance pro-tumor activities, contributing to the initiation and progression of liver cancer (Fig. 2). However, given the diversity of metabolites and nutrients, the precise impact of their depletion on immune cells in PLC requires further elucidation.

### The effect of liver cancer metabolites on immune cells

#### Lactate

The well-known “Warburg effect” rearranges glucose metabolism to produce excessive lactic acid, which has emerged as an important regulator in promoting immune evasion in PLC [134, 135]. For instance, upregulated MCT4 expression in HCC cells contributes to lactate exportation and subsequent TME acidification, ultimately leading to CD8 + T-cell exhaustion and M2φ polarization [136–138]. Therefore, targeting MCT4 can reinvigorate anti-tumor immunity in HCC and may be a promising therapeutic strategy to improve the efficacy of ICB-based therapy. In vitro, lactate also strengthens Treg cell functionality through Lys72 lactylation on MOESIN. This contributes to the activation of TGF-β signaling and the expression of key transcription factor FOXP3. Considering the critical role of Treg cells in immunotherapy, lactylation of MOESIN in Treg cells may predict the response of anti-PD-1 therapy in HCC [139]. Mitochondria are involved in cancer energy metabolism, and mitoribosome defects in HCC have been associated with an aggressive phenotype. Mechanistically, hepatic mitoribosomal defects induce elevated reactive oxygen species (ROS) and lactate production to circumvent a hostile environment for cytolytic T cells [140]. Lenvatinib, a multi-kinase inhibitor approved for systemic first-line treatment of HCC [141], promotes neutrophil recruitment by inducing CXCL2 and CXCL5 secretion in the TME. Simultaneously, tumor-derived lactate induces programmed cell death ligand 1 (PD-L1) expression in infiltrated neutrophils through the MCT1/NF-κB/COX-2 pathway, thus counteracting the efficacy of lenvatinib monotherapy in HCC mouse models [142].

#### Lipid

In addition to glucose metabolism and lactate, anti-liver cancer immunity is also largely dismantled by lipid metabolism and its products. Aberrant lipid metabolism in MASLD promotes hepatocarcinogenesis partly through intrahepatic CD4 + T-cell deprivation. Mechanistically, MASLD-associated linoleic acid production predominantly causes the accumulation of mitochondrial-derived ROS in mouse models and human samples. This mediates selective loss of intrahepatic CD4 + T lymphocytes due to their great mitochondrial mass [143]. Sirtuin 5 (SIRT5) is a metabolic regulator that removes succinyl, malonyl, and glutaryl groups from the lysine residues of mitochondrial and peroxisomal metabolic enzymes. Sun et al. reported that SIRT5 expression is repressed in tumor cells, which leads to aberrant BA biosynthesis in the peroxisome and subsequent M2φ-induced immunosuppression in oncogene-induced HCC mouse models [144]. PGE2, a bioactive lipid generated from arachidonic acid, has recently been implicated in immune evasion through multiple mechanisms [145]. In HCC cells, higher expression of COX-2, the rate-limiting enzyme of the PGE2 production, induces M2φ polarization to inhibit CD8 T-cell function in a multi-cellular co-culture system [146]. Recently, we also found that *KRAS* mutations upregulate COX-2 expression to promote PGE2 production in vitro. This results in an immunosuppressive TME dominated by excessive neutrophil infiltration and contributes to poor prognosis in iCCA [147].

#### Amino acids and adenosine

Other metabolites, such as amino acids and adenosine, also play pivotal roles in investigating the immune-suppressed TME. Intracellular arginine concentrations directly determine the metabolic fitness and functionality of activated T cells [100]. Chronic viral infection activates hepatocyte-intrinsic type I interferon (IFN-I) responses to break the urea cycle, leading to decreased arginine/ornithine ratios in the circulation and subsequently suppressed virus-specific CD8 + T-cell responses in chronic lymphocytic choriomeningitis virus (LCMV)-infected mice [148]. Other amino acid metabolisms, such as the methionine recycling pathway, have also been reported in HCC immune modulation. S-adenosylmethionine (SAM) and 5-methylthioadenosine (MTA) are two critical metabolites for methionine salvage. They promote T-cell exhaustion and exert significant impacts on HCC progression in both human samples and mouse models [6]. In HCC cells, hypoxia contributes to adenosine accumulation and extracellular secretion. This results in adenosine-mediated immunosuppressive roles on T cells and myeloid cells [149, 150]. Tumor cell-derived adenosine also synergizes with autocrine granulocyte–macrophage colony-stimulating factor (GM-CSF) secreted from activated TAM to promote their proliferation, thus maintaining the Mφ pools in HCC [151]. Collectively, the metabolic by-products produced by dysregulated cancer cells could directly play a profound role in immune cells within the TME (Fig. 2).

### Dysregulated PLC metabolism acts as signaling molecules regulating the TME

#### Hypoxia

**Fig. 3:**
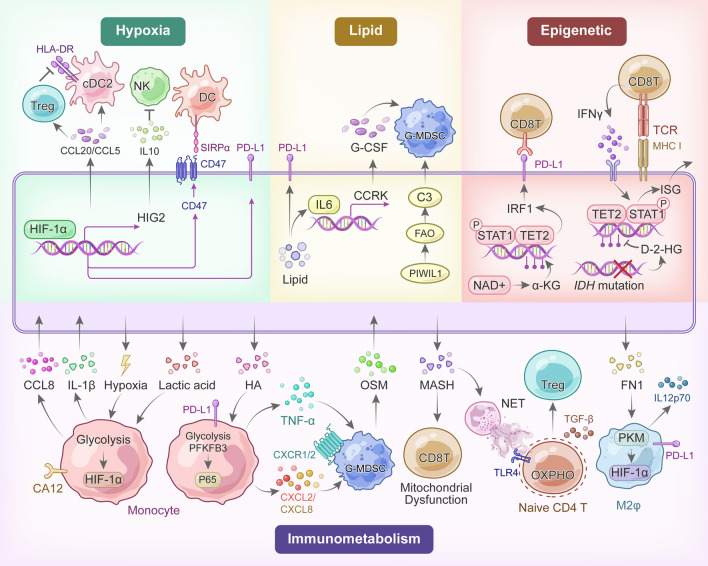
Metabolic signaling-mediated immune escape in tumor cells and immune cells. HA: hyaluronan; OSM: oncostatin M; NET: neutrophil extracellular traps; FN1: fibronectin 1; αKG: α-ketoglutarate; and D-2-HG: D-2-hydroxyglutarate

Metabolic reprogramming in PLC cells can also impact the TME through signaling molecules. Chen et al. proposed that sorafenib treatment increases intra-tumoral hypoxia, promoting immunosuppression through the stromal cell-derived factor 1α (SDF1α)-CXCR4 axis-induced Treg cells/M2φ accumulation in orthotopic HCC mouse models [152]. In hypoxia-high HCC regions of patient samples, the upregulation of tumor-derived chemokines, such as CCL20 and CCL5, leads to excessive Treg cell and cDC2 infiltration. Subsequently, in vitro assays demonstrated that the direct interaction of infiltrated Treg cells with cDC2 mediates HLA-DR loss, a critical antigen presentation molecule required for anti-tumor T-cell activation [153]. Additionally, in HCC patient samples, hypoxia-inducible gene 2 (HIG2), a HIF-1 target gene, fosters IL-10 secretion by HCC cells. This suppresses NK cell cytotoxicity through the activation of the STAT3 signaling pathway in co-culture assays [154]. Another study showed that hypoxic TME drives NK cell loss and dysfunction via mTOR-GTPase dynamin-related protein 1 (Drp1) mitochondrial fragmentation, leading to HCC immune evasion in human liver cancer and mouse liver models [106]. On the other hand, hypoxia often promotes tumor cell phagocytosis via CD103 + DCs, further recruiting and activating anti-tumor NK cells. However, tumor cells upregulate the innate immune checkpoint CD47 under hypoxic conditions, counteracting NK cell-mediated cytotoxicity with the “do not eat me” signal. Thus, blocking CD47 on the cell surface enhances NK cell-mediated anti-tumor immunity in the hypoxic microenvironment of HCC [155]. In iCCA, hypoxic surroundings can induce HIF1α and its downstream PD-1/PD-L1 pathway, creating an immunosuppressive TME [156]. Due to the crucial role of hypoxia in regulating intratumor immune components, targeting hypoxia depletion synergistically with current therapies (like TKIs plus ICBs) is warranted to advance the systemic treatment of unresectable HCC (Fig. 3).

#### Lipid

In addition to hypoxia, lipid metabolism is closely involved in the signaling molecules between PLC cells and the TME. Compared with HBV-HCC, MASLD-HCC is associated with a high incidence of mutations in *CTNNB1* over *TP53*. This shift in the driver mutations results in immune exclusion through the repression of the TNF receptor superfamily member 19 (TNFRSF19)-mediated senescence-associated secretory phenotype (SASP), as shown in a syngeneic immunocompetent mouse model [157]. Apart from MASLD, HCC patients with etiologies of HCV infections or alcohol abuse also harbor unique genetic variations, which may impact the metabolic reprogramming and the TME [75, 158]. Multi-omics study showed lipid accumulation in HCC elevates PD-L1 expression, inducing an immunosuppressive TME [28]. Due to excessive lipid accumulation, obesity heightens the risk of HCC, particularly in men, though the underlying molecular mechanism remains unclear [159, 160]. In MASH-related HCC mouse models, the androgen receptor (AR)-driven oncogene, cell cycle-related kinase (CCRK), combined with obesity-induced IL-6/STAT3 signaling, induces lipid metabolic reprogramming and MDSC-dominated immunosuppression [161]. Tumor cells increase Piwi-like RNA-Mediated Gene Silencing 1 (PIWIL1) to boost oxygen consumption and energy production through fatty acid metabolism, advancing HCC progression. Meanwhile, PIWIL1 regulates the secretion of Complement C3 to mediate interaction between HCC cells and MDSC, promoting the immunosuppressive cytokine IL10 in the TME [162]. Concerning the crucial role of lipid metabolism in the TME, serum lipids can predict the efficacy of anti-PD-1 therapy in iCCA patients, with apolipoproteinA-1 (APOA1) and triglycerides (TG) as notable independent predictors [163] (Fig. 3).

#### Epigenetics

Metabolic alterations accompanying hepatocarcinogenesis may also prompt epigenetic reprogramming of immune cells through the accumulation of epigenetically regulated metabolites. For instance, NAD + metabolism triggers PD-L1 expression on tumor cells, impairing the cytolytic activity of PD-1 + T cells through αKG-mediated epigenetic modifications [164]. In iCCA, *IDH* mutations produce the oncometabolite D-2-HG, suppressing the TET2-dependent epigenetic response to CD8 T-cell-derived IFN-γ in tumor cells. Consequently, the *IDH1/2* inhibitor AG120 synergizes with ICBs for advancing immunotherapy in the treatment of mIDH1-driven genetically engineered mouse models [165]. In a phase 3 clinical trial for mIDH1 iCCA, Ivosidenib/AG120 was found to significantly improve the progression-free survival (PFS) of patients compared with placebo, although the absolute improvement in the median PFS appeared modest [166, 167] (Table 3). These findings underscore that metabolic reprogramming of tumor cells acts as signaling molecules, contributing to immunosuppressive pathway deregulation in PLC (Fig. 3). Therefore, targeting these molecules is a viable treatment option for related metabolic diseases.

**Table 3:** Clinical trials on metabolic targets in PLC

Pathways	Targets	Compounds	Combination therapies	Clinical trials	Curative status	Aims (references)
Glycolysis	MCT1	AZD3965		NCT01791595	Phase 1 (Completed)	Advanced cancer [168]
	GLUT1	Aspirin	Lamivudine	NCT01936233	Phase 3 (Unknown)	Liver cancer after radical surgery
	PDK	Dichloroacetate		NCT00566410	Phase 1 (Completed)	Recurrent and/or metastatic solid tumors [169]
TCA cycle	Mitochondrial complex I	Metformin	Celebrex	NCT03184493	Phase 3 (Unknown)	HCC recurrence after hepatic resection [170, 171]
			Sirolimus	NCT02145559	Phase 1 (Completed)	Advanced solid tumors
			Vitamin C	NCT04033107	Phase 2 (Recruiting)	Malignant tumors
			Sorafenib	NCT02672488	Phase 3 (Unknown)	Advanced HCC
			Chloroquine	NCT02496741	Phase 1b (Completed)	*IDH1/2*-mutated malignant tumors (including CCA)
	IDH1/2	AG120 (Ivosidenib)		NCT02989857 NCT06081829 NCT05876754	Favorable OS benefit (Phase 3 completed and Phase 2/3b recruiting)	Previously treated patients with nonresectable or metastatic CCA /Advanced solid tumors (including CCA) [172–174]
			Gemcitabine and cisplatin	NCT04088188	Phase 1 (Active)	
			Nivolumab Ipilimumab	NCT05921760	Phase 1/2 (Recruiting)	
				NCT02073994	Phase 1 (Active)	
FAO and cholesterol metabolism	HMG-CoA reductase	Pravastatin	Sorafenib	NCT01075555 NCT01357486 NCT01418729 NCT01903694	Unfavorable OS benefit (Phase 3/2/2/3, completed)	HCC [175−176]
		Atorvastatin		NCT03024684	Phase 4 (Recruiting)	HCC recurrence after curative treatment
		Simvastatin		NCT02968810	Phase 2 (Active)	HCC in patients with cirrhosis
		Statin		NCT03490461	Observational	HCC recurrence after liver transplantation
	SPHK2	ABC294640	Hydroxychloroquine	NCT03377179	Phase 2 (Completed)	Advanced, unresectable CCA [178]
				NCT01488513	Phase 1 (Completed)	Advanced solid tumor (including HCC)
Amino acid metabolism	Glutamine	DRP-104	Durvalumab	NCT06027086	Phase 1b/2 (Not yet recruiting)	Advanced fibrolamellar HCC
	Glutaminase	CB-839	Standard chemotherapy	NCT02071862	Phase 1 (Completed)	Advanced and/or treatment-refractory solid tumors

### Metabolic reprogramming of immune cells diminishes anti-tumor immunity

Recent advances in cancer immunology have highlighted metabolic fuels/nutrients as a fourth signal beyond the three-signal model for effective T-cell priming and differentiation [179]. In the context of tumor-dysregulated metabolism, tumor-infiltrating immune cells inevitably experience metabolic stress. Consequently, they adapt metabolic characteristics to fulfill their duties [19]. In MASH-associated HCC mouse models, neutrophil extracellular traps (NETs) interact with naïve CD4 + T cells to facilitate its mitochondrial OXPHOS through TLR4 expression, contributing to their differentiation into immunosuppressive Treg cells [180]. Following anti-PD1 treatment, MASH also promoted aberrant activation of PD-1 + CD8 + T cells, leading to tissue damage, immune anergy, and reduced response to immunotherapy in preclinical HCC models [181]. Another study using multiple murine MASH models elucidated that MASH also impaired the mitochondrial fitness and motility of tumor-infiltrating CD8 + T cells. This impairment diminishes the efficacy of ICB therapy, which could be salvaged by metformin [182]. There are currently several clinical trials assessing the efficacy of metformin in treating HCC (Table 3). Meanwhile, gastrointestinal IgA^+^ metabolically activated B cells can license auto-aggressive T cells, promoting HCC development in an antigen-independent manner in MASH-induced HCC mouse models [183]. Of note, high serum cholesterol levels drive NK cell accumulation and subsequent lipid raft formation, further enhancing the anti-tumor activity in both DEN-induced HCC mouse models and Hepa1-6 (mouse hepatoma) subcutaneous models [184]. Due to the differences between the mouse models, the impact of lipid metabolism reprogramming on the TME remains controversial and requires further exploration.

In addition to immune-activated cells, immunosuppressive cells in liver cancer also undergo metabolic reprogramming. Chen et al. proposed that tumor-derived hyaluronan (HA) fragments induce aerobic glycolysis in monocytes and upregulate PD-L1 expression through the PFKFB3-NF-κB pathway [185]. Notably, the same group further illustrated that glycolytic monocytes in HCC produce large amounts of chemokines, such as CXCL2 and CXCL8. These chemokines induce neutrophil infiltration, while glycolytic monocytes secrete TNF-α to seduce oncostatin M (OSM) production from accumulated neutrophils. These processes ultimately lead to the metastasis of HCC, as shown by both ex vivo and in vitro experiments [186]. The acidic TME also induces a metabolic switch in monocytes to produce tremendous amounts of CCL8. This production promotes epithelial-mesenchymal transition (EMT) and HCC metastasis [187]. On the other hand, macrophage polarization is tightly correlated with metabolic rearrangement [188]. In mouse and human HCC, the serine/threonine kinase RIPK3 is downregulated in macrophages, leading to FAO-dominated M2φ polarization via the ROS/Caspase1/PPAR pathway [189]. M2-like TAMs upregulate the glycolysis pathway under hypoxic TME and produce excessive IL1β to facilitate EMT and subsequent metastasis in HCC [190]. Furthermore, the PKM2/HIF-1α axis in human HCC samples and syngeneic mouse models drives fibronectin 1 (FN1) production to instigate pluripotent polarization of macrophages, concurrent with anti-tumorigenic IL-12p70 production from glycolytic macrophages [191].

In this section, we discussed the metabolic interactions between liver cancer cells and immune cells (Fig. 3), highlighting a promising field for liver cancer treatment in the future. Some clinical trials have been conducted to assess the targeting of metabolic changes in the treatment of PLC (Table 3); however, combined targeting of these metabolic alterations using ICB should be specifically considered as this would significantly reinvigorate the anti-tumor immune response and thus achieve better therapeutic effects.

### Impact of gut microbiota-derived metabolites on the liver cancer microenvironment

The gut and the liver are physiologically connected due to their unique anatomical location. This gut–liver axis executes critical functions in nutrient metabolism and bacterial metabolite clearance, mainly through the portal vein [192]. Emerging studies have addressed the pivotal roles of the gut microbiota in PLC pathogenesis and anti-tumor therapy [5, 193–196]. The gut microbiota is closely associated with immunity and metabolism, underscoring their vital role in health and disease. The interaction between intestinal microbiota-secreted metabolites, including short-chain fatty acids, BA, indoles, and ethanol, and the PLC microenvironment requires further exploration [197]. In mice exposed to chemical carcinogens, obesity increases the levels of deoxycholic acid (DCA), a secondary bile acid produced by gut bacteria such as *Clostridium cluster XI* and *XIVa* strains. Through the gut–liver axis, DCA induces SASP in hepatic stellate cells (HSC) to trigger the secretion of various inflammatory factors, thus facilitating HCC progression [198]. Using 16S rRNA sequencing and serum metabolomic analysis, another independent group found that the development of MASLD-HCC induced by a high-cholesterol diet in mice was closely correlated to dysbiosis of the gut microbiota and the resultant alterations in metabolites [199]. The gut microbiota has also been implicated in HCC induced by high dietary intake of fructose. Investigation of the underlying mechanism showed that microbiota-derived acetate enhanced UDP-GlcNAc biosynthesis and O-GlcNAcylation, and hyper-O-GlcNAcylation of eukaryotic elongation factor 1A1 (eEF1A1) at T279 promoted the proliferation of tumor cells and HCC progression in the DEN + CCl_4_-induced HCC mouse model [200]. Butyrate is mainly produced by the gut microbiota during fermentation and is subsequently absorbed by the liver via the gut–liver axis [201]. Butyrate accumulation disrupts intracellular calcium homeostasis and induces the production of ROS, thus improving the efficacy of TKI therapy in HCC mouse models [202]. PSC and colitis, two well-known risk factors for iCCA, promote the exposure of gut-derived bacteria and lipopolysaccharide to the liver. This induces TLR4-dependent CXCL1 expression in hepatocytes, leading to the recruitment of CXCR2 + PMN-MDSC, and ultimately promoting an immunosuppressive TME [203]. Multi-omics studies incorporating 16S rRNA MiSeq sequencing in different cohorts from various regions of China have also identified gut microbes as non-invasive biomarkers for the early diagnosis of PLC [204–206].

Increasing evidence suggests that the gut microbiota modulates immune responses in the TME of PLC [5, 194, 203, 207, 208]. The liver immune system precludes gut-derived microbes and corresponding metabolites without evoking a systemic immune response, thus demonstrating immune privilege [209, 210]. Within intestinal microbiota, gram-positive bacteria predominantly convert immunostimulatory/primary into immunosuppressive/secondary BA. These recirculate to the liver through enterohepatic circulation. Secondary BA suppresses the recruitment of CXCR6 + tumorolytic NKT cells into the liver by reducing CXCL16 expression in liver sinusoidal endothelial cells (LSECs) [211–213]. Considering the impact of primary and secondary BA balance on the TME and PLC therapy, Ji et al. developed a strategy targeting BA receptors via nanoparticle-based delivery of modulators. This approach effectively reverses the immune privilege of HCC [214]. Bile acid metabolites such as isoalloLCA and isoDCA promote Treg cell differentiation through FOXP3 induction, while 3-oxoLCA and isoLCA inhibit Th17 cell differentiation by targeting RORγt in vitro and in vivo [215–217]. Thus, the role of the bile acid metabolic pathway in Treg/Th17 cell balance and anti-tumor immunity in the liver cancer microenvironment remains to be explored.

The gut microbiome strongly influences the tumor immune microenvironment and immunotherapy response [218–223]. Lipoteichoic acid (LTA), an obesity-induced gram-positive gut microbial metabolite, elevates COX-2 expression in the DCA-induced senescent HSCs. This leads to an immunosuppressive TME through PGE2 production in obesity-associated HCC mouse models [198, 224], potentially causing ICB resistance [225, 226]. Innate lymphoid cells, including ILC1, ILC2, and ILC3 subsets, are now recognized as crucial in tumor regulation by releasing specific cytokines. Hu et al. found a significant reduction of *Lactobacillus reuteri* in gut microbiota of mice with HCC. This leads to decreased short-chain fatty acid secretion, particularly acetate. The lack of acetate in TME weakens ILC3 anti-tumor functionality by increasing IL17A production. Thus, combining acetate supplementation with PD-1 blockades significantly boosts anti-tumor immunity [227]. Additionally, gut microbiome-derived metabolite D-lactate can shift TAMs from the M2 to M1 phenotype, remodeling the immunosuppressive TME in HCC mice [111, 228]. Lee et al. suggested that *Lachnoclostridium*, along with ursodeoxycholic and ursocholic acids, produce better responses to ICB treatment in patients with unresectable HCC [229].

People often consume dietary supplements for health benifits [230]. However, highly refined fermentable fibers may promote cholestasis and the development of HCC. In wild-type mice, a diet rich in inulin-enriched soluble fiber leads to microbiota-dependent cholestasis, hepatocyte death, and subsequent neutrophilic inflammation in the liver, culminating in HCC [231]. Ex vivo studies showed that gut bacteria convert dietary fiber into short-chain fatty acids in MASLD-HCC, leading to an immunosuppressive TME with an elevated CD4 + Treg cell/CD8 + T-cell ratio [208]. In conclusion, we discuss mechanisms by which the gut microbiota-related metabolites affect liver cancer TME (Table 4). Harnessing the gut microbiome could offer novel therapeutic strategies that target liver metabolism for PLC treatment.

**Table 4:** Gut–liver axis and immunoregulation of PLC

Microbiome	Metabolites	Target TME components	Function	References
Gram + bacteria (*Clostridium* spp)	Primary BA	LSEC	IS	[211–213]
	Secondary BA	NKT cells		
Gram + bacteria (*Phylum firmicutes*)	DCA	HSC	IS	[198, 224]
	LTA	CD8 + T cells		
*Lactobacillus intestinalis*	D-lactate	Macrophage	IS	[111, 228]
*Clostridium* spp.	Fermentation metabolites (BA)	Neutrophil	IS	[231]
*Bacteroides* spp.	SCFA (Butyrate, acetate, formate)	CD8 + T cells	IS	[208]
*Ruminococcus* spp.		APC		
*Veillonella* spp.		Treg cells		
*Clostridium* spp.				
*Lactobacillus reuteri*	Acetate	ILC3	IA	[227]

*BA* bile acid; *DCA* deoxycholic acid; *LTA* lipoteichoic acid; *SCFA* short-chain fatty acid; *LSEC* liver sinusoidal endothelial cells; *HSC* hepatic stellate cells; *IS* immunosuppression; and *IA* immune activation

## Conclusions

The cancer type and location influence nutrient availability and subsequent metabolism within the TME. Since the liver is the largest metabolic organ, the initiation and progression of liver cancer disrupt the metabolic homeostasis of the TME. In recent decades, there has been limited progress in the use of compounds targeting specific metabolic alterations in the treatment of PLC, likely due to metabolic plasticity, drug specificity, and the heterogeneous metabolic microenvironment [127]. The term “tumor metabolism” is used to indicate a common set of metabolic alterations accompanying malignancies. However, tumors are metabolically heterogeneous, mainly due to the complex cellular composition of the TME, including tumor, immune, and stromal cells. As both tumor cells and their surrounding anti-tumor immune cells share certain metabolic activities, agents designed to block these metabolic pathways in tumor cells may impair the proliferation, activation, and function of cytolytic immune cells, ultimately leading to unfavorable side effects. Thus, investigation of the mechanism involved in tumor immune metabolic crosstalk may help identify novel therapeutic targets. These less toxic and more specific metabolic targets could improve the unsatisfactory response rates of current therapies.

The role of the gut microbiome in health and disease has seen increasing research attention recently. Gut microbial dysbiosis promotes hepatocarcinogenesis by altering metabolic programs within TME. Exploring the metabolic connections among liver tumor cells, the gut microbiome, and immune cells may shed light on how to harness the gut microbiome to enhance current treatment strategies, particularly immunotherapies.

Many current studies have focused on animal models, while data derived from multi-omics analysis of clinical samples are generally descriptive and lack further validation. There is also a need for in-depth mechanistic studies to establish causalities between metabolites in the TME and liver cancer outcomes. Given that perturbations of metabolic processes and the gut microbiome are generally influenced by confounding factors, such as diet, environment, and host genetics, study of diverse cohorts from patients of different demographics, ethnicities, and geographical regions will be needed to determine the broader implications of the gut microbiome in PLC pathogenesis.

## Data Availability

Not applicable.
